# Comparative genome analysis of the first *Listeria monocytogenes* core genome multi-locus sequence types CT2050 AND CT2051 strains with their close relatives

**DOI:** 10.3934/microbiol.2022006

**Published:** 2022-03-21

**Authors:** Ogueri Nwaiwu

**Affiliations:** School of Biosciences, University of Nottingham, Sutton Bonington Campus, LE12 5RD

**Keywords:** *Listeria monocytogenes*, pan-genome, antimicrobial resistance genes, plasmid, bacitracin genes, genomics

## Abstract

Genome sequences of the three strains of *L. monocytogenes*, which are the first core genome multi-locus sequence types (cgMLST) 2050 and 2051 were reviewed and compared with 21 close relatives and reference genomes. Using a pan-genomic approach to analyse whole genome sequences, it was found that the strains consisted of approximately 2200 shared genes and a much greater pool of genes present as an accessory genome. An unknown transmissible sequence of approximately 91 kb harbouring bacitracin resistance genes found in strain LmNG2 (1/2b) was revealed to be an Inc18 plasmid. The CT2051, strain LmNG3 (1/2a) haboured more unique genes (252 vs 230) than the well-known reference strain LmEGD-e (1/2a). More studies to monitor new strains can help reduce food-borne outbreaks.

## 1. Introduction

*Listeria monocytogenes* is a food-borne pathogen that causes listeriosis disease and the infection poses a great threat to the health of human beings and animals. It persists in animals and food [1], which allows the organism to cross-contaminate and readily transmit between hosts. The impact on food safety is a concern [2] and attachment in minimal growth conditions found in processing facilities is aided by cellular components which are phospholipid in nature [3]. Food-borne outbreaks have been reported around the world and recently a devastating biggest outbreak of listeriosis in human history happened in South Africa between 2017 and 2018. That outbreak produced the biggest fatalities and hospitalizations from listeriosis ever known to humanity.

In the last decade, there has been an increase in the number of species of *Listeria* from 8 to 26 and they are found in different environments and geographical locations. Three strains of *L. monocytogenes* were isolated from tropical vegetables after which the molecular serotypes and evolutionary lineages based on *prfA* virulence gene cluster were determined [4]. Two of the strains LmNG1 and LmNG2 were later sequenced as types ST5 whereas strain LmNG3 belonged to ST155 [5]. Although these strains were not designated as new species, they were found to be new core genome sequence types. Strains LmNG1 and LmNG2 were designated cgMLST 2050 whereas strain LmNG3 were designated cgMLST 2051 after extensive comparisons with other strains worldwide [6]. The genome assemblies were deposited in GenBank and designated as new cgMLST [5].

Analysis of genomes can be used to learn more about horizontal gene transfer and other evolutionary trends [7]. To gain further insights, a pan genomic approach was used to closely compare the new core genome sequence strains with close relatives, known reference genomes (EGD-e, 10403S) in literature [8] and other published strains especially from the main *L. monocytogenes* (1/2a, 1/2b, 4b) molecular serotypes [9],[10] from other geographical regions around the world. This was to ascertain if there are emerging unknown trends in the evolution of the organism after comparison with older strains and new genomes recently released.

## 2. Materials and methods

### 2.1. Strains and genome selection

Sequences of strains selected for analysis were retrieved from the NCBI database with sequence accession or project numbers (Table 1) for review. The genome sequences used were chosen because they were assumed to be of reasonably good quality and were obtained from strains sourced from different geographical locations. More importantly, they were found to be close relatives of the three strains under study based on percentage identity after a search.

**Table 1.** Whole genome sequences of *L. monocytogenes* used in this study showing the accession numbers, strain names, Genome size/*N*50 contig length, country and source extracted from GenBank.

	*L. monocytogenes* strains	Sequence Accession	Genome size; *N*50 contig length (kb)	Country	Source
1	LmNG1 (1/2b)	FWPO0100000	2,968; 412	Nigeria	Vegetable
2	LmNG2 (1/2b)	FWPR0100000	3,060; 237	Nigeria	Vegetable
3	LmNG3 (1/2a)	FWPS0100000	2,933; 510	Nigeria	Vegetable
4	EGD-e (1/2a)	NC_003210.1	2944; -	France	Collection
5	10403S (1/2a)	NC_017544	2903; -	U.S.A.	Clinical
6	SLCC2540 (3b)	NC_018586	2976; -	Germany	Collection
7	H34 (1/2b)	CP020774	2892; -	Uruguay	Clinical
8	F8027 (4b)	MNCA01000000	2988;129	U.S.A.	Celery
9	OLM 10 (4b)	MIMA01000000	2925;143	U.S.A.	Clinical
10	MQ130033	MVEF01000000	2901; 352	Ireland	Human blood
11	MQ150004	MVET01000000	2932; 382	Ireland	Human placenta
12	SHL12-22	LRTX01000000	3015; 506	China	Food
13	SHL13-12	LRTY01000000	2986; 524	China	Food
14	10-092876-1063 (4b)	CP019616	2913;	Canada	Spinach
15	10-092876-1016 (1/2b)	CP019624	2965;	Canada	Meat
16	10-092876-1235 (1/2a)	CP019621	2966;	Canada	Spinach
17	10-092876-0168 (1/2b)	CP019615	3072;	Canada	Sprout
18	CFSAN002256	MJSI00000000	3012; 351	China	Collection
19	HM00108598	NZ_QEXK01000000	3017; 93	South Africa	Food
20	N16-0716	NZ_QELV00000000	2964; 344	Switzerland	Salad
21	FSL J2-064	NZ_AARO00000000.2	2828; 8	U.S.A.	Collection
22	NI3-0836 (1/2b)	NZ_RDSU01000023	3060; 482	Switzerland	Meat
23	C1-387	NC_021823	2988; -	-	-
24	Finland_1998	NC_017547.1	2874; -	-	Finland

### 2.2. Pan-genomic analysis

Using the whole genome sequence data of the strains of interest namely LmNG1, LmNG2, LmNG3, comparisons were made with the well-characterized *L. monocytogens* strains EGD-e and Lm 10403S among other whole genomes (Table 1). The default settings of the Roary software package [11] was used for pan genomic analysis and data obtained were processed or summarised with custom R code. Strains were compared to each other using Sourmash, a program that applies K-mer methods to estimate sequence similarity between sequence data sets quickly and accurately [12]. The Jaccard distance matrix for the strains, produced by the Sourmash compare method, was visualised as a dendrogram with the hclust function.

### 2.3. Unknown plasmid characterisation

The assembly graph for an unknown plasmid in strain LmNG2 was produced with the Spades program [13] and visualised with Bandage software [14] to show a putative plasmid. The blast ring image generator (BRIG) was generated as previously described [15]. A search was then carried out on Plasmid Finder [16] for identification after which a search on GenBank was carried out to establish the spread of the unknown plasmid.

## 3. Results

**Figure 1.**
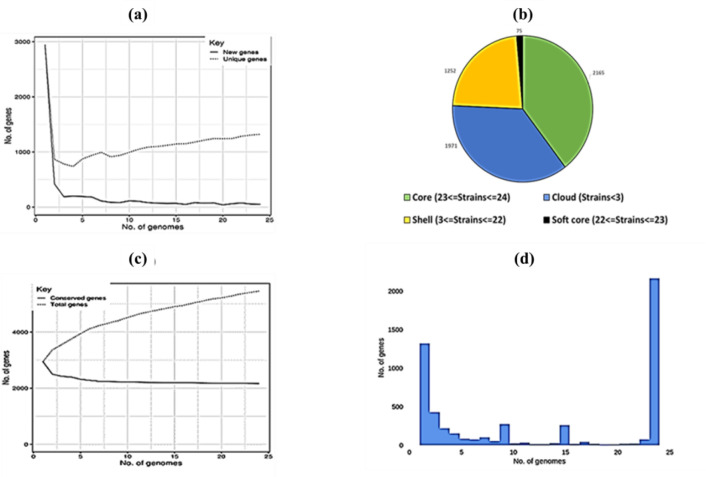
Analysis of *L. monocytogenes* genomes. Panels include new and unique genes (a), pan genomic pie (b), total and conserved genes(c), and genes distribution (d).

Previous studies of the pan-genome of *L. monocytogenes* brought new insights into intraspecific niche expansion and genomic diversification [17],[18]. Pan-genomic analysis is now very important to the understanding of the trends in emerging bacteria [19]. Using this approach, the selected strains were analysed. The relationships is presented in Figure 1. Figure 1a shows the description of new and unique genes, which is how the pan genome varies as genomes are added (in random orders). It was found that for these strains, aside from the core genome, the pool of additional genes are widely distributed and not concentrated in particular groups of strains. The pan genomic pie (Fig. 1b) describes the breakdown of core, soft-core, shell, and cloud genes among the strains with most strains possessing the core genes and less than three strains harbouring cloud genes. Figure 1c highlights conserved and total genes and shows that the core genome consists of approximately 2200 shared genes but that there is still a much greater pool of genes present as accessory genome. The histogram (Figure 1d) describes the frequency of genes and the number of genomes that share them. The bimodal distribution showed that most of the variable regions are in most of the strains and 1300 genes occur in single strains.

**Figure 2.**
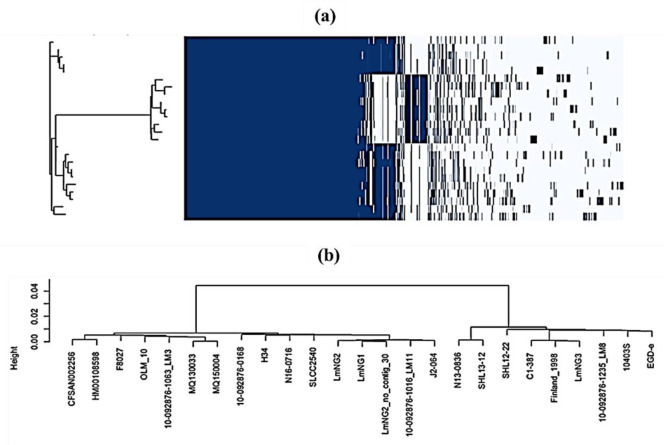
Pangenomic matrix (a) and a cluster dendrogram of strains analysed (b).

The pan genome matrix (Figure 2a) gives an overview of genes that are shared between strains indicating gene groupings present in different strain clusters. The matrix, which is like a heat map, scours the presence and absence of core (left hand side) and accessory genes (right hand side). The included ‘Tree’ is a clustering of the strains based on gene presence/absence. The estimation of similarity between sequence data sets (Figure 2b) indicates that LmNG1 and LmNG2 (both serotypes 1/2b) were in one cluster or monophyletic group whereas LmNG3 are grouped with strains Lm 10403S and EGD-e (all 1/2a serotype).

Data generated for gene groups present in the sequences analysed show that overall, some putative and hypothetical proteins are present in LmNG1-3 strains and absent in others strains analysed and vice versa. The maltose transport system permease protein MalG, and the maltose transport system permease protein MalF, a putative maltose ABC transporter, a sugar-binding protein and a transcriptional regulator DegA were detected in strain LmNG3 but not in the well known strain EGD-e. In contrast, the acyl-carrier-protein reductase FabK, the secretion machinery protein EssD, and TatD family deoxyribonuclease were detected in strain EGD-e but not in strain LmNG3. A comparison of close strains in the same clade (Figure 2b) highlighted that the LmNG1 strain has fewer unique genes than LmNG2. It was found that LmNG1 and LmNG3 had more unique genes than other strains they were compared with. In particular, LmNG3 had more unique genes than the widely studied strains EGD-e. Another comparison to establish present and missing genes between the new strains LmNG1-3 and their closest relatives in the same clade was performed. It was established that the group of genes that encode for the product threonine-phosphate decarboxylase and cystathionine gamma-synthase were observed to be absent in strains LmNG1 and LmNG2 but present in LmNG3. The analysis carried out showed that a group of genes that code for a tetracycline transcriptional regulator were present in strains LmNG1 and LmNG2 but absent in strain LmNG3. Three strains that formed a distinct monophyletic group namely LmNG3 (CT2051,) C1-387, and Finland_1988 (Figure 2b) all lacked a general stress protein sequence.

**Figure 3.**
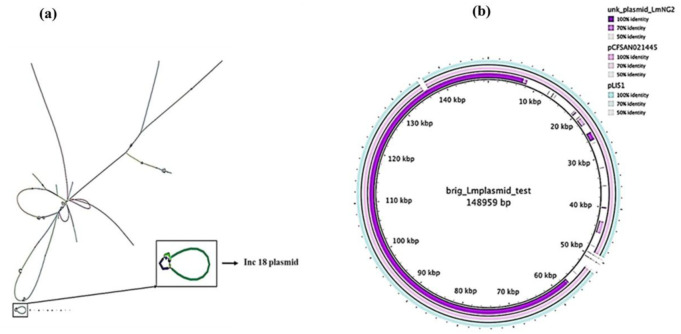
Putative plasmid regions separate from the main genome graph (a) and a BRIG construction for homologues of the ≈90 Kb plasmid sequence (b).

An unknown plasmid sequence was detected in strain LmNG2 (Figure 3a). The plasmid sequence resolved together separately from the rest of the genome scaffolds in the assembly graph and appears to be flanked by insertion elements. These scaffolds had a noticeably higher read depth than the median when assembled. There was an indication of the presence of bacitracin genes (*bcrB, bcrC*), which was linked to reference sequences annotated with these gene names in the RefSeq database. The (BRIG) diagram (Figure 3b) illustrates the similarity between LmNG2 (CT2050) and the closest strains harbouring the unknown plasmid. The read mapping and coverage showing boundaries reveal a degree of variation.

When a search was carried out on the Plasmid Finder database, the unknown sequence was revealed to be an Inc18 plasmid. The alignment was 100% with only another unnamed plasmid found in *L. monocytogenes* strain N1-011A plasmid that was sourced in the environment [20]. The sequence had high-scoring segment pair (HSP) perfect score (1650/1650) because it covered the entire length of the plasmid in the database. A BLAST search in Genbank with the sequences of the unknown plasmid produced top six hits with good per cent identity matching plasmids from different *Listeria* species that included *L. monocytogenes, L. welshimeri, L. grayi and L. innocua*. Sequence coverage score data of *L. monocytogenes* strains homologues ranged from 89–99% whereas other *Listeria* species ranged from 36–89% indicating that prevalence of the complete plasmid sequence was more prevalent in *L. monocytogenes*.

## 4. Discussion

Strains designated close relatives were isolates that are the same molecular serotypes, had at least 99% identity homology when a search was carried out with the new sequence types under study, and also fell under the same monophylogenetic group after preliminary evolutionary evaluation. In Figure 2b, the closest relatives of isolates LmNG1 and LmNG2 were the strains 10-092876-1016-Lm11 and J2-064 whereas for LmNG3 it was C1-387 and Finland _1998. It is a known fact that the core genomes of *L. monocytogenes* are highly conserved. The number (approximately 2200) of core genes in the three cgMLST strains and other 21 genomes estimated in this study were within the range of a previous study [21] but slightly less than the 2439 present in 10 sequenced genomes they analysed. In that report, the previous sizes highlighted were 2330 and 2465 core genes. Another study of *L. monocytogenes* strains sequenced from food processing plants in Denmark isolated over 20 years found that 90 strains shared a core genome of 2,381 genes and Moura et al. [6] recorded a total of 1748 core genes. A number of factors may affect the evaluation of core genes. Interpretation can vary between laboratories [22] and lateral gene transfer in the soil may be affected by geography and phylogeny [23]. A recent study [24] explained that soil moisture among other factors like salinity concentration and molybdenum helps determine genome flexibility and ability to survive and exchange DNA in various environments.

The absence of threonine-phosphate decarboxylase and cystathionine gamma-synthase and the presence of a tetracycline transcriptional regulator in strains LmNG1 and LmNG2 (CT2050) and vice versa in LmNG3 (CT2051) suggests horizontal gene acquisition or loss. The genes are orthologous in bacteria because threonine-phosphate decarboxylase synthesises (R)-1-amino-2-propanol phosphate in *Salmonella enterica* [25], a Gram negative organism and cystathionine γ-synthase facilitates methionine biosynthesis in other microorganisms [26]. Tetracycline resistance genes have since been described in *L. monocytogenes* [27] and its transcriptional regulators are prevalent in Gram negative bacteria where they act as resistance determinants [28].

The absence of a stress response protein observed in LmNG3 (CT2051) and other strains in a distinct monophyletic group are not uncommon because novel proteins can be repressed or appear in different environmental conditions. No universal stress proteins are common to all hot or cold stress situation [29] and the pattern of expression of stress proteins will require monitoring to ascertain if there is a link between virulence and stress response in the new strains.

Plasmid-mediated resistance is very important in the spread of antimicrobial resistance in humans [30] and animals [31]. Classification is usually performed by the assignment of incompatibility which is based on the principle that plasmids cannot exist within a cell line for multiple generations when they compete for the same replication system [32]. The 90 kb plasmid sequence found in this study is within the range of putative plasmid contigs that are approximately 4 kb to 170 kb reported by Schmitz-Esser et al., [33] after a large scale survey of global dissemination of plasmids in *L. monocytogenes*. The occurrence, spread and transfer mechanisms of the Inc18 plasmid family found in this study has been elucidated by Kohler et al. [34]. In that report, It was highlighted that isolation was originally from a clinical *Streptococcus agalactiae* strain which later spread to the environment, waste water, and domestic animals. Also, members of this plasmid family are often detected in *Streptococci enterococci*, and are implicated in vancomycin resistance of *Staphylococcus*. The fact that this plasmid was detected in other *Listeria* species but not found in strain LmNG1 which is of the same molecular serotype and in the same monophyletic group with LmNG2 (Figure 2b) harbouring it, suggests that it is transmissible and was acquired from the environment. This phenomenon has been demonstrated by other investigators who showed that environmental plasmids can be transferred into foodborne pathogenic bacteria at high transfer ratios [35].

When new strains with unknown or specific attributes are discovered, researchers normally compare these strains with popular reference strains that are regularly used by others. Wang et al. [36] used the whole genome sequence of *L. monocytogenes* strain ST477 isolate from a frozen food sample and compared it with 58 other genomes. They found that changes in genes involved in multiplication and invasion were the differences between them. Also Kuenne et al. [37] sequenced novel plasmids from *L. monocytogenes* serotype 1/2c and 7 and compared them with 10 published *Listeria* plasmids and found a common evolutionary background. In other organisms, it was established that there were high similarities in two selected strains of *Lachancea thermotolerans* and the type strain CBS 6340T in gene content [38], and a comparative analysis of 24 strains of *Fructilactobacillus sanfranciscensis* showed that mapA and mapB genes responsible for maltose phosphorylase were present in only 10 out of the 24 genomes analysed [39].

Other workers have demonstrated that genome comparative analysis can identify antimicrobial resistance genes and give insights into genome evolution and stability. Stability and functional traits are usually carried out by targeting specific genes identified from genome comparisons of strains with unique genes. Heat-inactivated cells of *L. monocytogenes* caused a reduction of both fengycin and iturin amounts found in *Bacillus* sp. P34, isolated from a freshwater fish gut after comparative genome analysis [40]. In another functional and comparative analysis of essential genes [41], it was revealed that many basic physiological and biochemical processes such as transcription differ between *R. solanacearum* and other bacteria. These sorts of findings highlight the usefulness of whole-genome sequencing comparisons because they provided crucial genetic insights for a deeper investigation of intraspecific variability [38]. Huang et al. [42] have explained that these variations may be caused by environmental stress which can activate natural transformation.

The report of transmission and function of novel plasmids in *L. monocytogenes* is increasing. An investigation [43] of processing plants that make ready-to-eat seafood in France found that resistant genes to biocides were present in a 90.8 kbp plasmid. In that study, it was posited that this plasmid was identical to isolates from clonal complex CC204 and CC155, and it was concluded that the high similarity indicates lateral gene transfer among strains aided by recombination and deletion. A novel plasmid of similar size (91 Kb) capable of self transmission has been found by Mao et al. [44] to exist in *L. monocytogens* sequence types (ST) namely ST87, ST59, ST9 and ST120). An opportunity exists to explore the similarities of the two aforementioned plasmids with the one of similar size detected in this study. Another study [45] found a novel plasmid variant that was functional in cells of *L. monocytogenes* and was responsible for cadmium resistance.

Despite the report of the presence of these plasmids, *L. monocytogenes* is not presently known as a notorious organism that spreads anti-microbial resistance through plasmids and this could be due to a number of factors. It has been reported [46] that the ability to persist and transmit may be affected by the interactions of other genetic elements and the plasmid. The transmission and potential function of plasmids may also be aided by the interactions between plasmids. However, it has been highlighted that the rate of these associations is not high enough to ensure the persistence of plasmids [47]. Furthermore, it has been pointed out [48] that in bacteria genomes, positive selection may inhibit plasmid coexistence by retaining plasmids with benefits, and a consensus is that plasmid transmission is limited by fitness costs [49].

## 5. Conclusions

This review describes more features of the first core genome sequence types CT2050 and CT2051 of *L. monocytogenes*. Pan genomic comparisons revealed that some protein groups present in the new strains are absent in well-known strains and vice versa. The presence or absence of these proteins will require confirmation in the laboratory. The prevalence of the Inc18 plasmid family appear to be spreading in *L. monocytogenes*, hence there is a need for more studies to determine the antimicrobial resistance effects and any other environmental function the presence may convey on the organism.
